# A Novel Real-Time Detection and Classification Method for ECG Signal Images Based on Deep Learning

**DOI:** 10.3390/s24165087

**Published:** 2024-08-06

**Authors:** Linjuan Ma, Fuquan Zhang

**Affiliations:** 1 School of Computer Science and Technology, Beijing Institute of Technology, Beijing 100081, China; malinjuan@bit.edu.cn; 2 College of Computer and Control Engineering, Minjiang University, Fuzhou 350108, China

**Keywords:** artificial intelligence, deep learning, ECG images, detection and classification, real time

## Abstract

In this paper, a novel deep learning method Mamba-RAYOLO is presented, which can improve detection and classification in the processing and analysis of ECG images in real time by integrating three advanced modules. The feature extraction module in our work with a multi-branch structure during training can capture a wide range of features to ensure efficient inference and rich feature extraction. The attention mechanism module utilized in our proposed network can dynamically focus on the most relevant spatial and channel-wise features to improve detection accuracy and computational efficiency. Then, the extracted features can be refined for efficient spatial feature processing and robust feature fusion. Several sets of experiments have been carried out to test the validity of the proposed Mamba-RAYOLO and these indicate that our method has made significant improvements in the detection and classification of ECG images. The research offers a promising framework for more accurate and efficient medical ECG diagnostics.

## 1. Introduction

In recent years, artificial intelligence [1,2,3,4,5,6,7,8,9,10] in the electrocardiogram (ECG) has sparked intense interest in the medical community. A cardiac electrical activity recorder is a diagnostic and therapeutic device that can be used without invading the body. It monitors the electrical signals of the heart by installing sensors on the body surface. The electrical activity stimulated by the heartbeat is captured by ECG technology and converted into waveform patterns, including a P wave, QRS group, and T wave. These graphs can reveal the beating pattern of the heart, the condition of the myocardium, and the operation of the electrical signal conduction mechanism. Waveform analysis assists medical professionals in identifying a variety of cardiac conditions, such as cardiac arrhythmias, myocardial ischemia, and other cardiac structural abnormalities. There are also a variety of cardiac pathologies identified by ECG tracings, including atrial fibrillation, atrioventricular block, ventricular tachycardia, ventricular fibrillation, left ventricular hypertrophy, right ventricular hypertrophy, premature ventricular contractions, Wolff–Parkinson–White syndrome, long QT syndrome, and so on [11]. It can be clearly observed that the electrocardiogram is commonly used in medical practice as a preferred method for diagnosing and tracking cardiovascular disorders [12].

As ECG technology continues to evolve from its traditional analog form to its advanced digital form, its accuracy and utility have increased significantly. However, the old ECG analysis technology mainly relies on manual interpretation and empirical criteria, which has some obvious shortcomings [1,2,3,4]: (1) The interpretation of an electrocardiogram depends on the rich experience of doctors; traditionally, the interpretation of an electrocardiogram depends on the practical experience and profound disciplinary accomplishment of doctors. The accuracy of this strategy is influenced by the doctor’s personal practical experience and knowledge reserve, so it is more vulnerable to the influence of personal subjective judgment. In ECG interpretation, differences in expert opinion may lead to multiple interpretations of the meaning of the map, thereby increasing the likelihood of diagnostic errors or omissions. (2) The risk of misdiagnosis and missed diagnosis: due to subjective judgment, different doctors may reach different conclusions from the same ECG chart, increasing the risk of misdiagnosis and missed diagnosis. (3) Time-consuming manual interpretation: manually interpreting ECG charts takes a substantial amount of time, especially when handling large volumes of patient data, which can lead to increased workloads for doctors and affect diagnostic efficiency. (4) Limited accuracy: human analysis may not be able to track subtle fluctuations in the ECG signal, which in turn may lead to doctors ignoring early or insidious signs of heart disease. (5) Difficult data processing and lack of automation: The interpretation of ECG signals is complex and often performed manually, which results in inefficiencies in processing and interpreting these signals, and conventional ECG analysis technology lacks autonomous auxiliary tools, which makes it more difficult to process and interpret large quantities of data quickly. It creates difficult problems in the management and interpretation of ECG data. (6) Professional training needs and variable skill levels: Doctors require professional training to accurately interpret ECG charts, which increases the cost of medical resource training. Additionally, the varying skill levels among doctors can lead to inconsistent quality in diagnostic results.

To address the aforementioned limitations and challenges, and with the rapid advancement of artificial intelligence (AI) and deep learning technologies, leveraging AI in ECG analysis is crucial [5,6,7,8,9,10]. The application is critical to enhancing the identification and tracking of cardiovascular disease, helping to reduce the stress of the disease and improve the quality of life of patients. In the field of ECG analysis, intelligent systems exhibit a number of significant advantages. Firstly, through the application of deep learning architecture, such as convolutional neural network (CNN) and long short-term memory network (LSTM), AI can autonomously identify subtle features from large-scale datasets, which achieves high-precision identification in the analysis of electrocardiograms, thus promoting the accuracy of diagnostic results. The use of intelligent technology can ensure the standard of medical diagnosis and reduce the risk of diagnostic errors or omissions caused by the subjective judgment of doctors [5,6,7,8]. Secondly, AI technology can efficiently process and interpret large amounts of data in just a few seconds, greatly improving the timeliness of electrocardiogram (ECG) analysis, which plays a vital role in the clinical environment that requires a rapid response. Artificial intelligence technology can achieve continuous monitoring, instantly detect abnormal situations, and trigger prompts, so it shows excellent applicability in telemedicine and dynamic tracking. Thirdly, automated ECG analysis systems can reduce the reliance on specialist doctors, thereby lowering healthcare costs, particularly in regions with limited medical resources. AI can also process and analyze large volumes of historical ECG data to uncover potential disease patterns and trends, facilitating the development of personalized treatment plans. Finally, AI systems can integrate ECG data with other physiological signals and clinical data, offering a more comprehensive health assessment [7,8,9,10]. AI not only aids in diagnosis but also assists doctors in formulating treatment plans, thereby enhancing the overall quality of medical services.

In the literature, an increasing number of researchers have focused on the application of deep neural networks in ECG for analysis, detection, and prediction. The strategies are broadly categorized into three main categories, namely, convolutional neural networks (CNNs), long short-term memory networks (LSTMs), and convolutional long short-term memory networks (CNN-LSTMs). CNNs, as they are known, are a class of deep learning models that are specifically created to process data with grid morphological structures, such as images. CNNs, which are composed of convolutional layers, pooling layers, fully connected layers, and activation functions, are particularly effective for image analysis tasks due to their ability to capture spatial dependencies through the use of convolution operations. They play an important role in ECG analysis for feature extraction, which can automatically learn and extract relevant features from raw ECG signals, eliminating the need for manual feature engineering. By leveraging multiple convolutional layers, CNNs create hierarchical feature representations that capture both low-level and high-level patterns in ECG signals. They can not only be widely used to detect various types of arrhythmias from ECG data, but can also classify ECG segments into different categories, such as normal rhythm, atrial fibrillation, and other types of arrhythmias. For example, Baloglu et al. [13] proposed an end-to-end deep learning model based on a CNN on the standard 12-lead ECG signal for the diagnosis of MI. Labati et al. [14] proposed Deep-ECG to use a deep CNN for extracting significant features from one or more leads. Ullah et al. [15] presented an one-dimensional convolutional neural network that can accurately classify the electrocardiogram signal even in the presence of environmental noise. Ozaltin et al. [16] proposed a novel convolutional neural network (CNN) architecture to detect ECG types, which can also automatically extract features from images. Zhao et al. [17] presented a method based on wavelet transform combined with a deep convolutional neural network for automatic ECG classification. Jahmunah et al. [18] proposed the GaborCNN model for automated categorizing of diseases in ECG signals. Due to the time series nature of ECG signals, long short-term memory (LSTM) networks perform exceptionally well in ECG analysis, which are a special type of recurrent neural network (RNN) that excels at handling and predicting time series data. One of the key advantages of LSTMs is their ability to capture long-term dependencies, making them well-suited for processing complex time series signals. Jyotishi et al. [19] designed a new LSTM-based framework for person identification using an ECG signal. Kim et al. [20] proposed employing bidirectional LSTM-based deep recurrent neural networks (DRNNs) with late fusion to create a real-time system for ECG-based biometric identification and classification. Boda et al. [21] presented a novel framework for patient-specific electrocardiogram (ECG) beat classification using LSTM-based RNNs, featuring two LSTM models connected in parallel, each processing single-channel ECG input. In recent years, many researchers have attached much importance on CNN-LSTM in ECG analysis due to it capturing both local and global features, allowing the model to understand both the spatial and temporal aspects of ECG signals, leading to more comprehensive and accurate analysis. It can be applied in ECG classification and detection. For example, Petmezas et al. [22] initially extracted ECG features via CNN and then input to an LSTM model for temporal dynamics memorization to obtain more accurate classification into the four ECG rhythm types. Rai et al. [23] proposed an automated detection system for MI using electrocardiogram (ECG) signals through a CNN, a hybrid CNN-LSTM, and an ensemble technique to select the optimum performing model. Çınar et al. [24] presented a novel network based on Hybrid Alexnet-SVM for the classification of ECG signals. Sowmya et al. [25] used a CNN-LSTM method to study the classification of arrhythmia signals and ECG signals, which has a higher accuracy compared to CNN. Dey et al. [26] extracted 21 temporal features from 12-lead data to reduce redundancy and class imbalance while preserving vital information, and then fed these features into a detection model with a one-dimensional CNN and a bi-LSTM layer, classifying the data into HC, MI, and non-MI subjects.

Although the deep neural networks CNN, LSTM, and CNN-LSTM applied in ECG signals have achieved significant advancements, there are still some limitations in computational resources, real-time performance, and comprehensive feature extraction. Therefore, the You Only Look Once (YOLO) algorithm has been focused on ECG signals for classification and detection recently. It can process entire images in a single pass, making it highly efficient and suitable for real-time ECG analysis, the quick processing capability of which is critical for continuous monitoring and immediate diagnosis. Moreover, its architecture is streamlined and optimized for efficiency, reducing the complexity and computational overhead, which makes it easier to deploy on various hardware, including portable and edge devices. When applied in ECG signals, YOLO can capture comprehensive spatial features and context in the entire image, which is advantageous for identifying complex patterns and anomalies in ECG images. Hwang et al. [27] proposed a novel fast and simple arrhythmia detection algorithm based on YOLO to detect each heartbeat on long-duration ECG signals without R-peak detection and classify an arrhythmia simultaneously. Das et al. [28] used on YOLOv4 network to detect four classes of the proposed ECG images dataset. Jenifer et al. [29] proposed a novel deep learning-based YOLO-ECG model for ECG arrhythmia classification in portable monitoring, where denoised ECG signals are fed into the YOLO network with a Gaussian Error Linear Unit (GELU) activation function to detect ECG abnormalities. Ang et al. [30] leveraged a novel loss-modified YOLOv8 model for arrhythmia detection to categorize single-lead ECG signals, which was fine-tuned on the MIT-BIH arrhythmia dataset, enabling real-time continuous monitoring.

Great progress has been made in using deep neural networks in ECG signals; however, realizing the tradeoff between the speed and accuracy of the classification and detection of ECG signals is still a challenge. To address the problem, in this paper, a novel deep learning method Mamba-RAYOLO is proposed, which is based on a YOLOv9 network and can contribute to the classification and detection of signals in ECG images.

The contributions of our study are as follows:

- To enhance the initial feature extraction, RepVGG is incorporated into the backbone, which employs a multi-branch structure during training to capture a broader range of features. Then, it is reparameterized into a simpler form for efficient inference. The inclusion of RepVGG can contribute to capturing diverse and rich feature information from ECG images, which is crucial for accurately identifying subtle patterns and anomalies in the signals.
- In order to deal with the attention problem of space and channel, the BRA module is proposed. By dynamically capturing and paying attention to the key regions in the feature map, and using the region-level attention mechanism, the ability of the network to extract spatial features is optimized, while the interference of background noise is reduced.
- The C2f_VSS component is adopted to deepen and enhance the performance of structure diagram generation. The component combines the superior efficiency of feature extraction and the ability of spatial data processing, which significantly reduces the complexity of operation while ensuring a strong level of feature expression. Multiple nesting and accumulation are implemented, which enhances the integration of features, resulting in a more recognizable feature map in the ECG image classification task.

This paper is divided into five sections as follows. Section 2 introduces the Materials and Methods of the proposed network, including the architecture, the optimization modules and the dataset. Section 3 shows the Results of the proposed method in our work. In Section 4 and Section 5, the Discussion and Conclusions are given.

## 2. Materials and Methods

In this section, the proposed network framework is first introduced, followed by a description of each optimization module and the functions employed within the network structure, and a description of the dataset used in this study.

### 2.1. The Architecture of Mamba-RAYOLO

**Figure 1:**
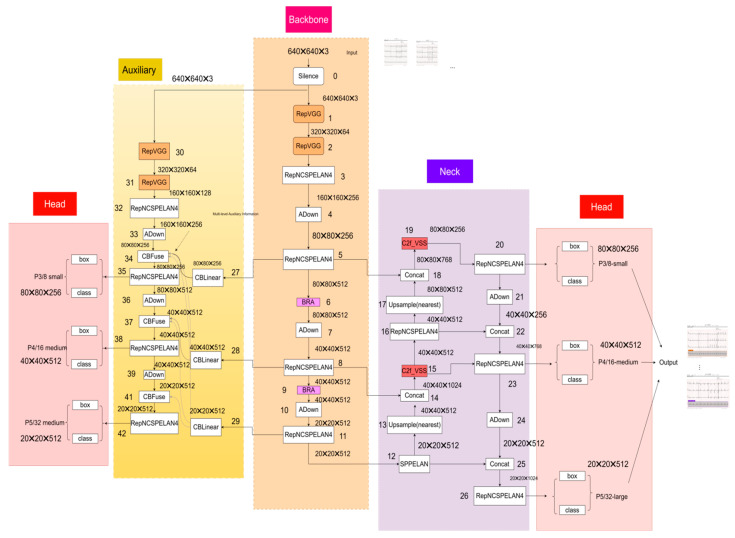
The architecture of Mamba-RAYOLO.

The architecture of Mamba-RAYOLO can be seen in Figure 1, and there are mainly four modules composed of, namely a backbone part, neck part, head part, and the Multi-Level Reversible Auxiliary Branch. When the data are input, they go through the backbone part, neck part, the Multi-Level Reversible Auxiliary Branch, and the head part in turn. The role of the backbone is to extract the basic features of the ECG images, which can help to more effectively analyze and understand important patterns and abnormalities in the ECG images, thereby improving the overall detection and classification performance. The role of the neck part is to further process and integrate the basic features extracted by the backbone to achieve specific task objectives. The head part is used to compute the output and predict the classification and location of the object, the sizes of the outputs of which are 80 × 80 × 256, 40 × 40 × 512, and 20 × 20 × 512, respectively, as shown in Figure 1. Furthermore, the Multi-Level Reversible Auxiliary Branch is an advanced module that can enhance feature extraction and representation capabilities.

#### 2.1.1. Backbone Part

In the backbone part of the proposed network, the process and step number of which can be seen in Backbone in Figure 1, when the ECG image data x are input to the Silence module, which acts as a placeholder or maintains data flow continuity, it can directly pass the input data to the output. The output data then enter the two RepVGG modules, as shown in step 1 and 2, where spatial features in the input feature map are extracted through channel splitting and convolution operations, enhanced by BatchNorm and activation functions to improve feature representation. Then the output data in step 2 are input into the RepNCSPELAN4 module in step 3, which performs initial convolution, channel splitting, feature map concatenation, and a final convolution to restore channel count. The function of the RepNCSPELAN4 module is to capture multi-scale feature information through multiple convolutional layers, thus enhancing network expressiveness, which effectively balances high-efficiency computation with high-quality feature extraction, integrating features of different scales to improve detection and classification performance. The output feature map then proceeds to the ADown module in step 4, which performs a series of pooling, splitting, convolution, and concatenation operations, reducing the spatial dimensions while retaining feature information, crucial for improving computational efficiency and reducing complexity. Subsequently, the output feature map is input into a RepNCSPELAN4 module, as shown in step 5 followed by the BRA attention mechanism in step 6. By introducing the BRA attention mechanism after the RepNCSPELAN4 module, it can act as an advanced attention mechanism to enhance feature representation, filtering out most irrelevant key-value pairs and retaining the most relevant parts, thereby improving computational efficiency. The resulting feature map is then fed into another ADown module in step 7, and then input into a RepNCSPELAN4 module in step 8, followed by the BRA attention mechanism in step 9. Then, it is input to the ADown and RepNCSPELAN4 modules again to produce the final output feature map in step 11. 

#### 2.1.2. Neck Part

In the neck part of the proposed network, as shown in Figure 1, the feature map which was obtained by RepNCSPELAN4 in step 11 of the backbone part is then fed into the SPPELAN module in step 12, where multi-scale features are captured through spatial pyramid pooling operations. Subsequently, the resulting feature map undergoes upsampling and feature fusion with the feature map from the backbone. The resulting data are then processed in the C2f_VSS module for convolution operations and VSS feature processing. The obtained data are fed into the RepNCSPELAN4 module for further feature extraction, resulting in the feature map. The operations of upsampling, feature fusion, C2f_VSS, and RepNCSPELAN4 are repeated. Then, the resulting feature map is downsampled and fused with the previous feature maps. Next, the resulting feature map undergoes repeated operations of RepNCSPELAN4, downsampling (ADown), and feature map fusion (Concat), resulting in the feature map. Finally, the obtained feature map undergoes RepNCSPELAN4 operations, as shown in step 26 of the neck part in Figure 1.

#### 2.1.3. Multi-Level Reversible Auxiliary Branch

In the proposed network, the Multi-Level Reversible Auxiliary Branch can significantly improve detection and classification performance by combining multiple feature maps and utilizing a reversible auxiliary branch mechanism. The role of CBLinear is to balance and adjust the channel contributions of the feature maps, optimizing feature representation through linear transformation. In this module, CBLinear operations are performed on the output data from the backbone, as shown in steps 27, 28, and 29. The input data undergo two RepVGG operations, one RepNCSPELAN4 operation, and one ADown operation, as shown in steps 30, 31, 32, and 33. The resulting feature map is fused with the output data of CBLinear using CBFuse. Subsequently, the resulting feature map is input into the RepNCSPELAN4, ADown, and CBFuse modules, repeated twice. Finally, the obtained feature map undergoes a RepNCSPELAN4 operation, which can be seen in step 42.

### 2.2. The RepVGG Module

**Figure 2:**
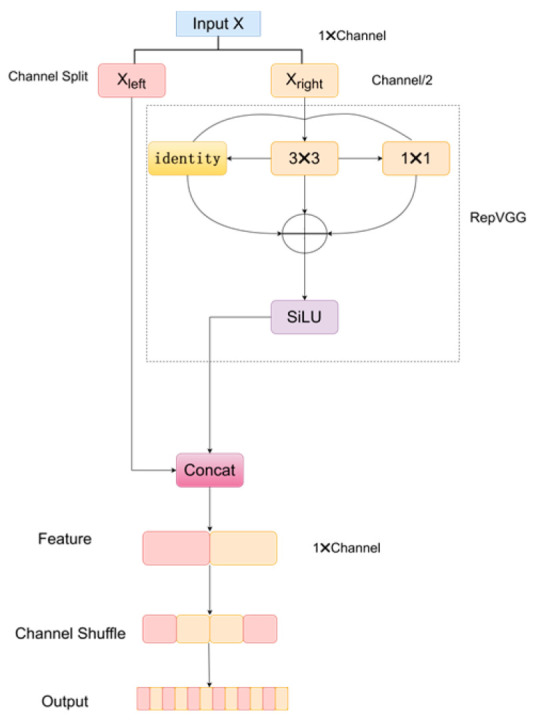
The RepVGG module.

In the backbone part of the proposed network, RepVGG [31] is adopted, which can be shown in Figure 2. Compared to Conv, there are several advantages of RepVGG. Firstly, the multi-branch structure of RepVGG can capture more feature information during training, enhancing feature extraction capability. Secondly, through structural reparameterization, RepVGG can maintain efficient computational performance during the inference stage, reducing inference time. Finally, the enhanced feature extraction capability helps to better recognize complex ECG features, improving detection accuracy.

Let the input feature map be X with dimensions (H, W, C). The channel splitting computation is shown in Equation (1), where X_left_ and X_right_ represent the front and back parts of the input feature map in the channel dimension, each with dimensions (H, W, C/2).

X_left_, X_right_ = split(X, dim = C/2), (1)

X_right_ is processed in the RepVGG module, including a 3 × 3 convolution as shown in Equation (2), a 1 × 1 convolution as shown in Equation (3), and an identity mapping as shown in Equation (4), where σ denotes the activation function. Then, the outputs of these three are summed, as shown in Equation (5).

Y_3×3_ = σ(Conv3 × 3(X_right_)), (2)

Y_1×1_ = σ(Conv1 × 1(X_right_)), (3)

Y_id_ = X_right_ (4)

Y_repvgg_ = Y_3×3_ + Y_1×1_ + Y_id_, (5)

X_left__and X_right_ are concatenated in the channel dimension, as shown in Equation (6). Next, channel shuffling is performed on the concatenated features, as shown in Equation (7). Finally, the shuffled features are used as the final output, as shown in Equation (8).

Y_concat_ = concat(X_left_, Y_repvgg_, dim = C), (6)

Y_shuffle_ = ChannelShuffle(Y_concat_), (7)

Output = Y_shuffle_, (8)

### 2.3. The RepNCSPELAN4 Module

**Figure 3:**
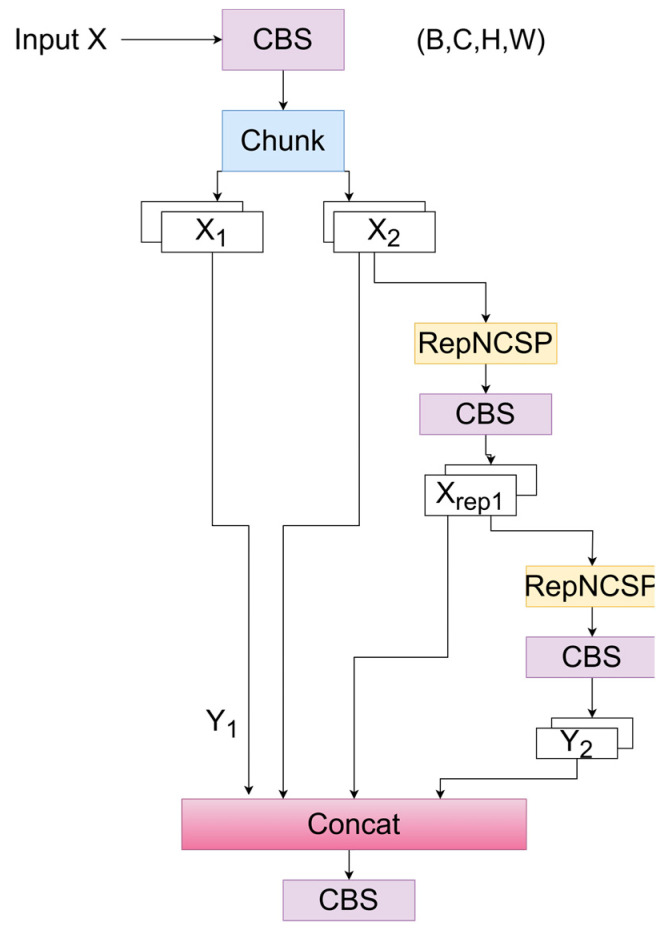
The RepNCSPELAN4 module.

In the backbone part of the proposed network, the RepNCSPELAN4 module is utilized, as shown in Figure 3. Different from RepVGG, which focuses primarily on extracting basic features, RepNCSPELAN4 can further refine and enhance these features. By first using RepVGG to extract initial features and then processing them with RepNCSPELAN4, the network can progressively refine the feature representations, ensuring richer and more discriminative feature information. As a result, the network can more comprehensively extract important features from ECG images, improving the overall detection and classification performance.

When inputting X ∈ $R^{B\times C\times H\times W}$, the module firstly performs a convolution operation on the input feature X, as shown in Equation (9). In this equation, Conv denotes the convolution operation, C represents the number of output channels, k is the kernel size, s is the stride, p is the padding, and σ represents the activation function.

X_conv_ = σ(Conv(X, C, k = 1, s = 1, p = 0)), (9)

The convolved feature map is split into channels, with each branch C/2 channels, as shown in Equation (10). The first branch is passed directly, as indicated in Equation (11). The second branch undergoes convolution and the activation function twice, respectively, as shown in Equations (12) and (13).

X_1_, X_2_ = split(X_conv_, dim = C/2), (10)

Y_1_ = X_1_, (11)

X_rep1_ = σ(Conv(X2, C/2, k = 3, s = 1, p = 1)), (12)

Y_2_ = σ(Conv(Xrep1, C/2, k = 3, s = 1, p = 1)), (13)

Concatenate Y_1_ and Y_2_ in the channel dimension to obtain Yconcat, as shown in Equation (14). Finally, pass the concatenated feature map Yconcat through a convolution layer and an activation function to obtain the output Y, as shown in Equation (15).

Y_concat_ = concat(Y_1_, Y_2_), (14)

Y = σ(Conv(Y_concat_, C, k = 1, s = 1, p = 0)), (15)

### 2.4. The SPPELAN Module

**Figure 4:**
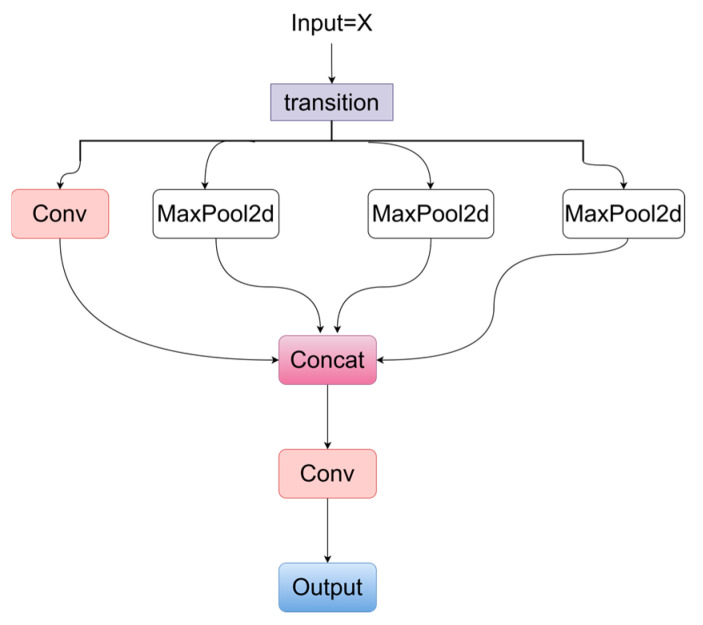
The SPPELAN module.

In the head part of the proposed network, the SPPELAN module shown in Figure 4 is used to extract the multi-scale global features through the operation of the space pyramid pool, and the features of different scales can be combined to enhance the ability of the model to capture features of different scales. The architecture of SPPELAN can be seen in Figure 4.

The input feature map is denoted as X, and the convolution operation is performed on the input feature map, as shown in Equation (16), where Conv represents the convolution operation, C represents the number of output channels, k is the size of the convolution kernel, s is the stride, p is the padding and σ represents the activation function.

X_trans_ = σ(Conv(X, C, k = 1, s = 1, p = 0)), (16)

Then, the input feature map is processed through four different branches. Branch 1 performs a convolution operation as shown in Equation (17), while branches 2, 3, and 4 perform max pooling operations, as shown in Equations (18)–(20), respectively. Next, the feature maps from the four branches are concatenated, as shown in Equation (21).

Y_conv_ = σ(Conv(X_trans_, C, k = 1, s = 1, p = 0)), (17)

Y_max1_ = MaxPool2d(X_trans_, k = 5, s = 1, p = 2), (18)

Y_max2_ = MaxPool2d(X_trans_, k = 9, s = 1, p = 4), (19)

Y_max3_ = MaxPool2d(X_trans_, k = 13, s = 1, p = 6), (20)

Y_concat_ = concat(Y_conv_, Y_max1_, Y_max2_, Y_max3_), (21)

Finally, a convolution operation is applied to the concatenated feature map to restore the number of channels, as shown in Equation (22). The final output feature map Y is the output of the SPPELAN module.

Y= σ(Conv(Y_concat_, C, k = 1, s = 1, p = 0)), (22)

### 2.5. The BRA Module

The Bi-Level Routing Attention (BRA) mechanism, when applied to ECG images, offers several advantages by incorporating dynamic sparsity and region-level attention computation. First, the BRA mechanism focuses on important regions within the feature map to enhance the quality of feature representation, which contributes to more accurately capturing key patterns and anomalies in ECG detection capabilities. Secondly, dynamic sparse computation allows the model to adaptively allocate computational resources based on the characteristics of the input data during inference. It can maintain efficient computation while concentrating on the most crucial regions for detection and classification to improve the efficiency and performance. Thirdly, the region-level attention mechanism enables the model to focus on specific local regions rather than the entire feature map. This is particularly important when dealing with ECG images, as it significantly reduces interference from irrelevant information, enhancing clarity and specificity. Finally, by concentrating on important regions and dynamically adjusting computational resources, the BRA mechanism improves the accuracy of detecting subtle and local features in ECG images. This is especially effective for identifying abnormal patterns in ECG signals, such as arrhythmias and myocardial infarctions.

The BRA module first partitions the input feature map X ∈ $R^{B\times H\times W\times C}$ according to regions, resulting in X′ ∈ $R^{B\times S^{2}\times \frac{HW}{S^{2}}\times C}$, as shown in Equation (23), where B represents the batch size, C represents the number of channels, H represents the height of the feature map, W represents the width of the feature map, and S is the side length of the region.

(23) $$X^{\prime }=\mathrm{patchify}(X,\;\mathrm{patch}\_\mathrm{size}=\frac{H}{S}),$$

Then, a linear transformation is applied to the queries, keys, and values, as shown in Equations (24)–(26), where $W^{Q}$, $W^{k}$, and $W^{V}$ are a projection matrix.

(24) $$Q=X^{\prime }W^{Q},$$

(25) $$K=X^{\prime }W^{k},$$

(26) $$V=W^{\prime }W^{V},$$

By averaging the queries and keys within each region, the region-level queries and keys are obtained, as shown in Equations (27) and (28).

(27) $$Q_{R}=\frac{1}{N}\sum_{i=1}^{N}Q_{i},$$

(28) $$K_{R}=\frac{1}{N}\sum_{i=1}^{N}K_{i},$$

The adjacency matrix $A_{R}$ of the region map is computed through the dot product of the region-level queries and keys, as shown in Equation (29).

(29) $$A_{R}=Q_{R}\cdot K_{R}^{T},$$

Based on the adjacency matrix $A_{R}$, the k most relevant regions are selected to obtain the index matrix $I_{R}$ of the relevant regions, as shown in Equation (30).

(30) $$I_{R}=\mathrm{topk}(A_{R},\;k),$$

Using the index matrix $I_{R}$, the key and value pairs within the regions are collected, as shown in Equations (31) and (32).

(31) $$K_{G}=\mathrm{gather}(K,\;I_{R}),$$

(32) $$V_{G}=\mathrm{gather}(V,\;I_{R}),$$

Token-to-token attention is computed, as shown in Equation (33).

(33) $$A=\mathrm{softmax}(Q\cdot K_{G}^{T}),$$

The output is calculated by weighted summation, as shown in Equation (34).

(34) $$O=\mathrm{Attention}(Q,\;K_{G},\;V_{G})+\mathrm{LCE}(V),$$

Finally, the output is restored to dimensions (H, W, C), as shown in Equation (35).

(35) $$\mathrm{output}=\mathrm{unpatchify}(O,\;\mathrm{patch}\_\mathrm{size}=\frac{H}{S}),$$

### 2.6. The VSS Block

**Figure 5:**
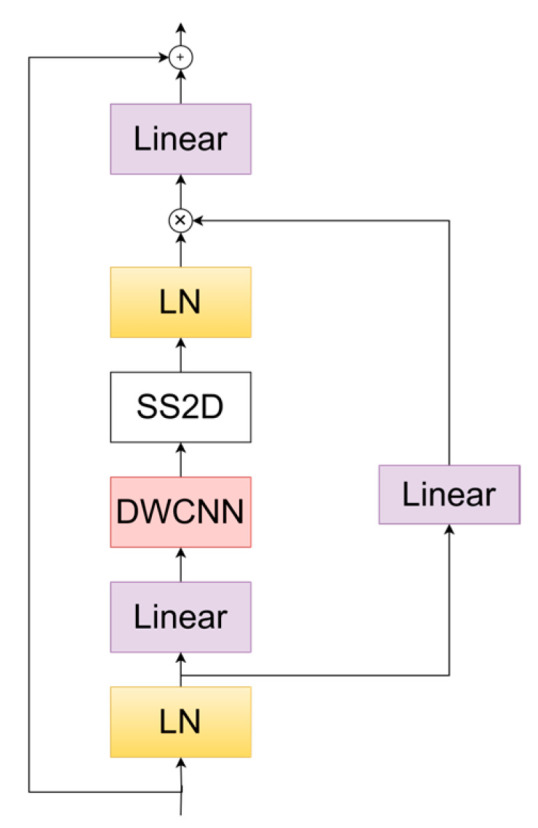
The VSS block.

The VSS block based on Visual Mamba [32] is utilized in our work, the architecture of which can be seen in Figure 5. In the VSS block, the input features first encounter a linear embedding layer, then split into dual paths. One branch undergoes depthwise convolution and SiLU activation. It continues to the SS2D module, followed by layer normalization, and merges with the residual stream after SiLU activation. Unlike typical vision transformers, this VSS block avoids positional embeddings and opts for a streamlined structure without an MLP phase, enabling denser block stacking within the same depth budget. 

In the linear layer, feature transformation and dimensionality reduction are performed. Through the linear layer, the number of channels in the feature map can be reduced, thereby decreasing computational load. Layer Normalization (LN) normalizes the feature map, stabilizing the training process and improving the network’s convergence speed. SS2D (Shifted Spatial 2D Convolution) is a shifted spatial convolution used to enhance spatial feature processing capabilities. The shifting operation allows for better capture of spatial information. Depthwise Separable Convolution (DWConv) efficiently extracts spatial features and reduces computational load. It consists of depthwise convolution and pointwise convolution, reducing computational complexity while maintaining feature representation capability. Element-wise Multiplication and Addition perform element-wise multiplication and addition on features from different paths, used for feature fusion and enhancement. This approach allows for better integration of features from different sources.

The input feature x and the hidden state are transformed through the linear layer, as shown in Equations (36) and (37).

(36) $$h^{\prime }=W_{1}h+b_{1},$$

(37) $$x^{\prime }=W_{2}h+b_{2},$$

The state transition is performed using LN and SS2D, as shown in Equations (38) and (39).

(38) $$h_{LN}=\mathrm{LayerNorm}(h^{\prime }),$$

(39) $$h_{SS2D}=\mathrm{SS}2D(h_{LN}),$$

The convolution operation is performed using DWConv, as shown in Equation (40), and the projection operation is performed through the linear layer, as shown in Equation (41).

(40) $$h_{DWConv}=\mathrm{DWConv}(h_{SS2D}),$$

(41) $$h_{linear}=W_{3}h_{DWConv}+b_{3}\;,$$

After multiple layers of computation, the final output feature is obtained, as shown in Equation (42).

(42) $$y=h_{linear}$$

### 2.7. Dataset

**Figure 6:**
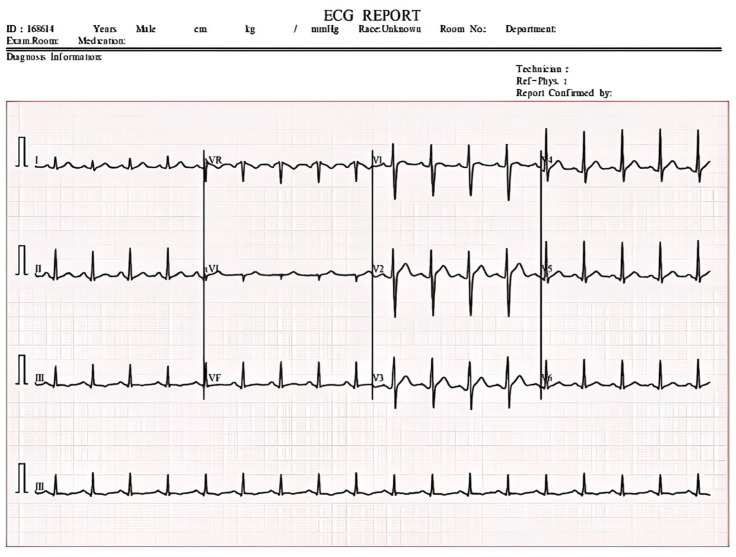
A sample of ECG raw data.

**Figure 7:**
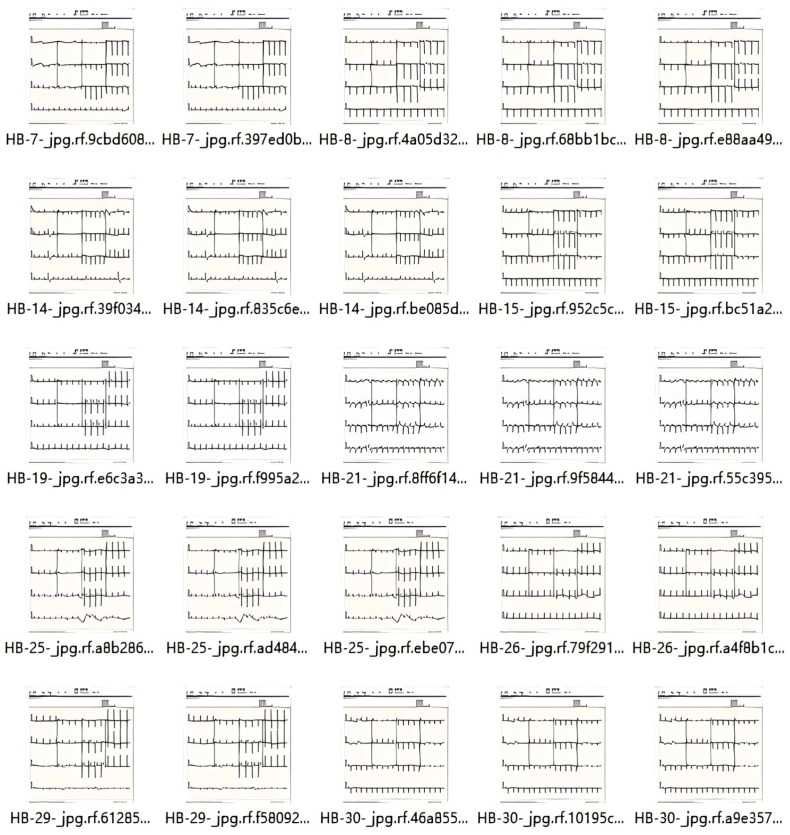
The ECG image dataset.

The ECG image dataset utilized in this work is sourced from the information from a total of 271 patients, which has been collected from the Cardiology Departments of Chittagong Medical College and Dhaka Medical College Hospital. A sample of ECG raw data can be seen in Figure 6 and the ECG image dataset containing several ECG images is shown in Figure 7. The dataset comprises 1231 images, categorized into four different classes, including ECG HB, history-MI, MI-ECG, and normal-ECG. HB stands for the ECG images of patients who have an abnormal heartbeat, MI stands for the ECG images of myocardial infarction patients, history-MI stands for the ECG images of patients who have a history of myocardial infarction, and normal stands for normal peoples’ ECG images. The dataset aims to help the scientific community to conduct research for all kinds of cardiac pathologies and cardiovascular diseases. 

The auto-orientation of pixel data and image augmentation were applied in the dataset, including geometric transformations, pixel transformations, random cropping and padding, and affine transformations, which help to increase the diversity of training data and improve the model’s generalization ability and robustness, and the images were resized to 640 × 640.

Many deep learning algorithms necessitate data annotation. Specialists assisted in completing the data annotation process. All ECG images were converted to the YOLO format. Using the data from the CSV file included with the dataset, the labeling software generates a text file for each image. In the YOLO format, a bounding box is defined with the features corresponding to a class.

## 3. Results

The proposed Mamba-RAYOLO was trained and tested with an NVIDIA GeForce GTX 3080ti 12 GB GPU with the Cuda version 11.7. During the training stage, the batch size is set to 4, the epoch 200, the learning rate 0.001, and the optimizer is Adam.

**Figure 8:**
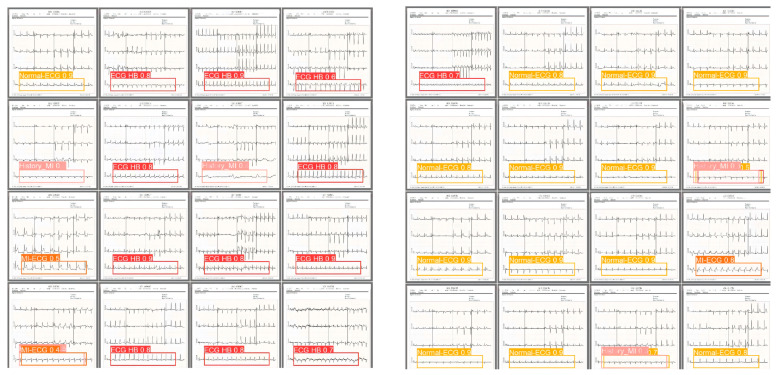
The results of the detection and classification of ECG images.

Figure 8 shows the results of the detection and classification of ECG images by the proposed Mamba-RAYOLO, which can detect four different classes, including ECG HB, history-MI, MI-ECG, and normal-ECG. 

**Figure 9:**
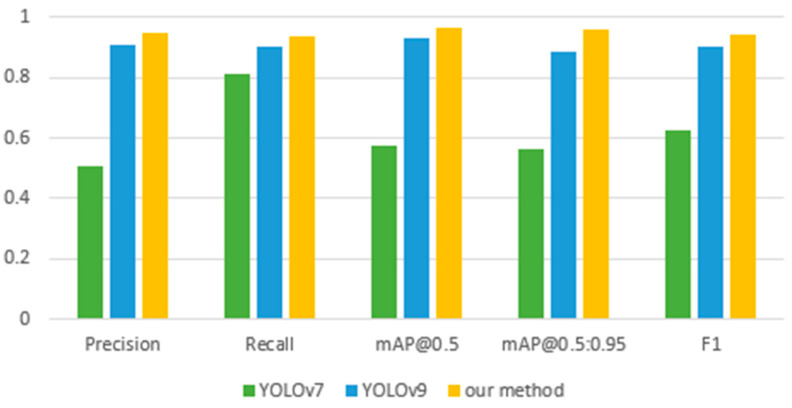
Performance comparison with other method.

**Figure 10:**
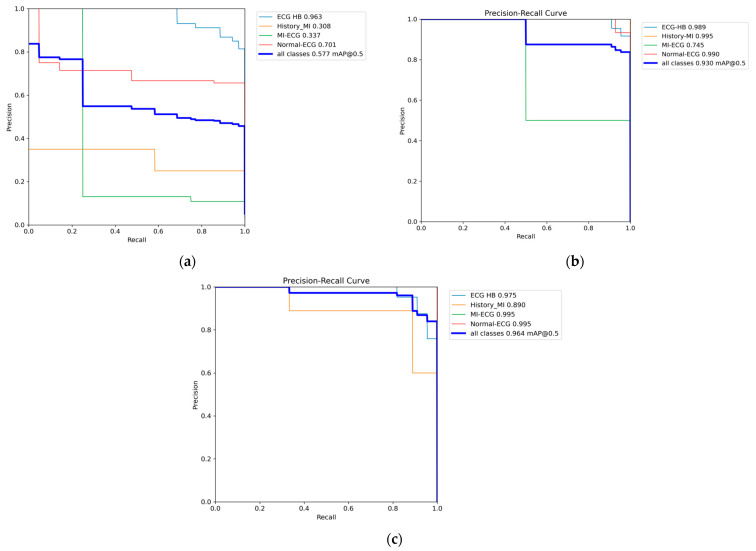
Comparison of PR. (**a**) Precision–recall curve of YOLOv7; (**b**) precision–recall curve of YOLOv9; (**c**) precision–recall curve of our proposed method.

**Figure 11:**
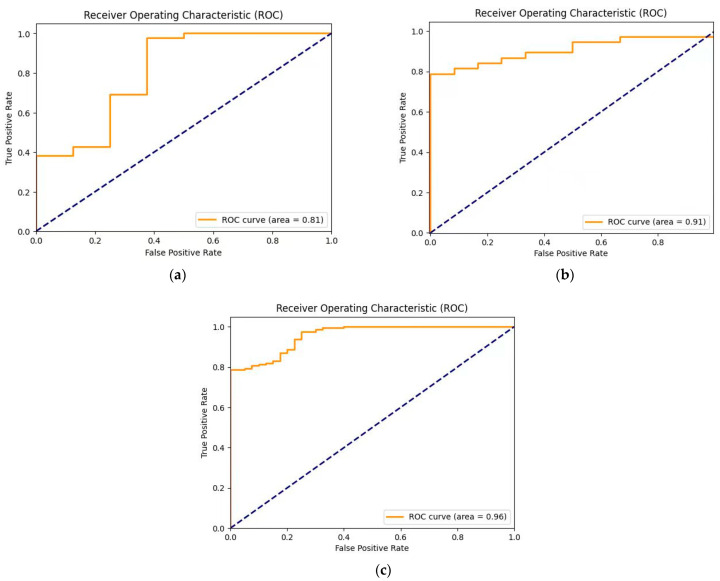
Comparison of ROC. (**a**) ROC of YOLOv7; (**b**) ROC of YOLOv9; (**c**) ROC of our proposed method.

Table 1 shows the performance comparison of YOLOv7, YOLOv9, and the proposed Mamba-RAYOLO, which is based on YOLOv7, and contains the metrics of precision, recall, mAP_50_, and mAP_50:95_. It shows that our proposed method is more accurate than the others, with precision of 0.947, recall 0.939, mAP_50_ 0.964, and mAP_50:95_ 0.959. The GFLOPs (Giga Floating-point Operations Per Second) of Mamba-RAYOLO in our study is the highest at 337.7G, namely, this means that the GPU has the strongest ability to handle floating-point arithmetic, and has better performance when performing tasks with a large number of calculations. The bar chart of the performance comparison is shown in Figure 9. Table 2 shows the ablation study of each method in the proposed Mamba-RAYOLO. It demonstrates that RepVGG, BRA, and C2f_VSS all improve the precision and accuracy of Mamba-RAYOLO. The comparison of PR and ROC is shown in Figure 10 and Figure 11, respectively. It can be clearly seen from Figure 10 that the area under the PR curve of our proposed algorithm is larger than the others, indicating the best model performance. In the ROC curve, the AUC value of our proposed algorithm is 0.96, higher than that of other algorithms, demonstrating that the proposed algorithm has the best classification performance.

**Figure 12:**
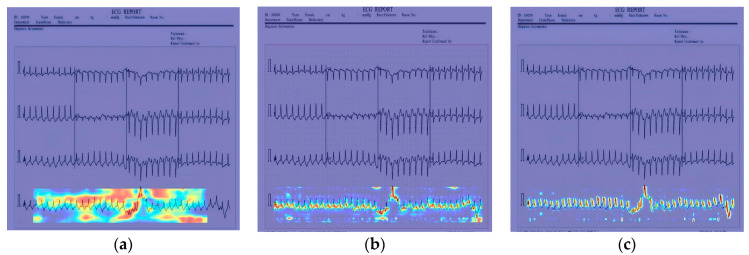
Comparison of feature visualization of ECG ID:168500. (**a**) Feature visualization of YOLOv7; (**b**) feature visualization of YOLOv9; (**c**) feature visualization of our proposed method.

**Figure 13:**
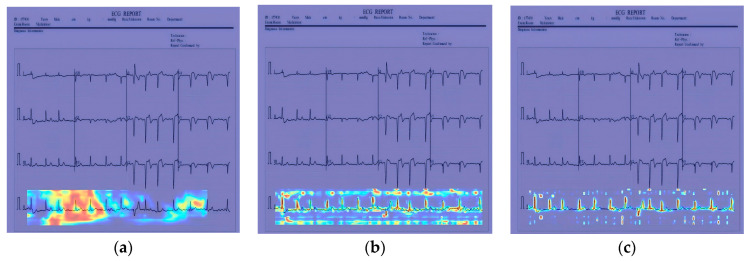
Comparison of feature visualization of ECG ID:177430. (**a**) Feature visualization of YOLOv7; (**b**) feature visualization of YOLOv9; (**c**) feature visualization of our proposed method.

**Figure 14:**
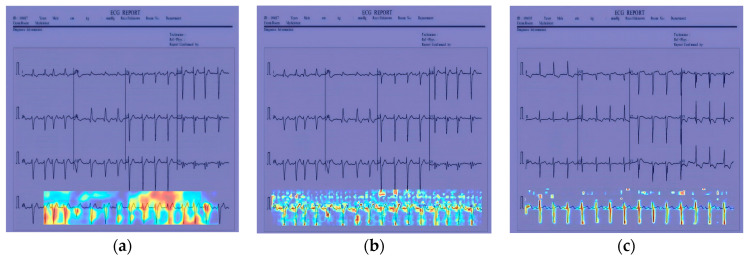
Comparison of feature visualization of ECG ID:168605. (**a**) Feature visualization of YOLOv7; (**b**) feature visualization of YOLOv9; (**c**) feature visualization of our proposed method.

**Figure 15:**
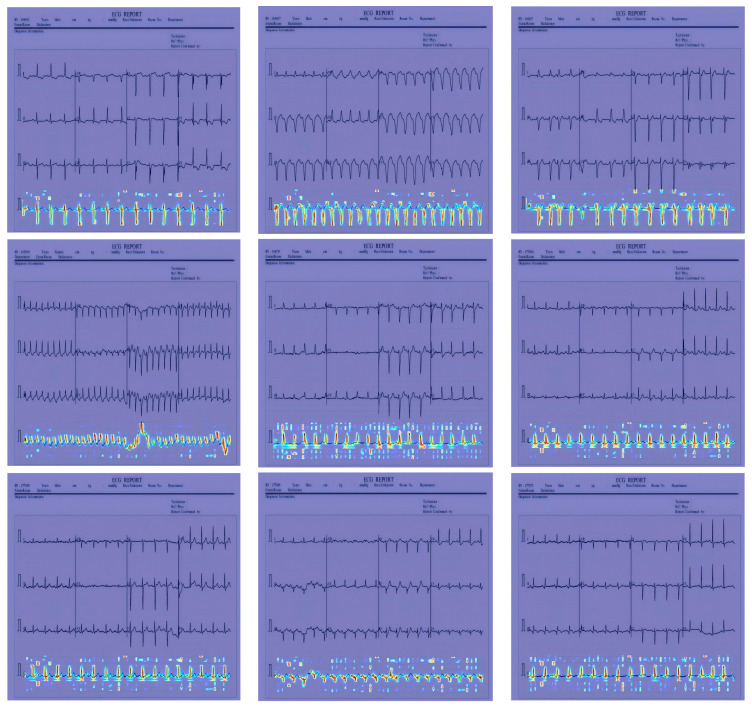

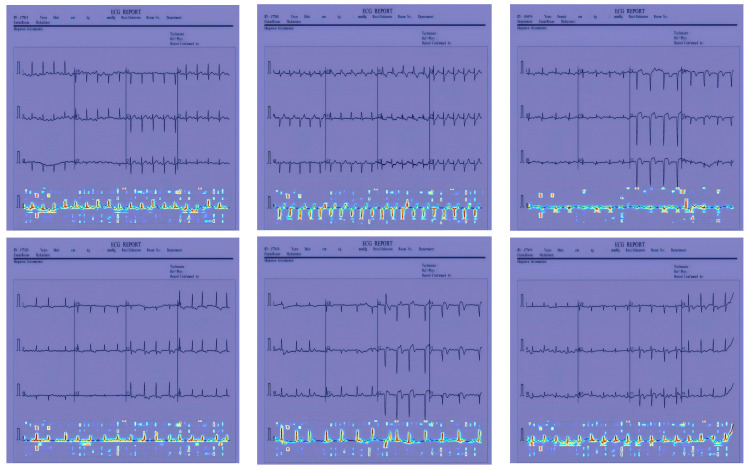
Feature visualization of ECG data with our proposed method Mamba-RAYOLO.

In Figure 12, Figure 13 and Figure 14, the performance comparison of the feature visualization of ECG data using YOLOv7, YOLOv9, and our proposed method Mamba-RAYOLO is shown. The ECG data were selected randomly from different categories, and ECG ID:168500, ECG ID:177430, and ECG ID:168605 were selected. When dealing with ECG image feature visualization and heat map generation, compared with YOLOv7 and YOLOv9, it can be seen that the use of our proposed method Mamba-RAYOLO has a clearer contour and contrast, which is more able to highlight the key features of ECG signals, the data are more complete and comprehensive, and it has the strongest robustness and best performance compared with other methods. Figure 15 shows the feature visualization effect on ECG data with our proposed method Mamba-RAYOLO, which can capture the ECG signal data comprehensively and accurately, highlight the key features, there is no information loss phenomenon, and the color distribution is clear.

## 4. Discussion

These results suggest that the integration of RepVGG, BRA, and C2f_VSS modules can significantly enhance the YOLOv9 architecture’s ability to process and analyze ECG images. It is understandable because the RepVGG multi-branch structure during training captures diverse features, while BRA’s attention mechanism focuses on critical regions, and C2f_VSS refines these features through efficient convolution operations and robust feature fusion. Collectively, these enhancements lead to improved detection and classification performance, making the network more adept at identifying complex patterns and anomalies in ECG signals. This study demonstrates the effectiveness of combining these advanced modules to achieve superior performance in medical image analysis tasks, which is consistent with the theory. Despite the great advantages mentioned above, there are still some limitations in speed and efficiency, which remain to be solved in order to improve and optimize the speed in the proposed network.

## 5. Conclusions

In this study, the Mamba-RAYOLO architecture integrating the RepVGG, Bi-Level Routing Attention (BRA), and C2f_VSS modules is presented to improve the performance when processing and analyzing ECG images. Each of these advanced modules contributes uniquely to the overall capabilities of the network:

1. RepVGG: By incorporating RepVGG into the backbone, it can leverage its multi-branch structure during training to capture a wide range of features. This reparameterization approach ensures efficient inference while maintaining rich feature extraction, which is critical for identifying subtle patterns in ECG images.
2. Bi-Level Routing Attention (BRA): The addition of BRA allows the network to focus dynamically on the most relevant spatial and channel-wise features. This selective attention mechanism enhances the network to accurately detect and classify important regions within the ECG images, thereby improving detection accuracy and computational efficiency.
3. C2f_VSS: By utilizing C2f_VSS, the features extracted by the backbone can be further refined to enable efficient and effective spatial feature processing. The element-wise operations for feature fusion further enhance the discriminative ability of the feature maps.

The combined effect of these enhancements has led to significant improvements in the network to process and classify ECG images. The enhanced architecture demonstrates superior performance in detecting and identifying complex patterns and anomalies in ECG signals, which is essential for accurate medical diagnoses.

In conclusion, this study highlights the effectiveness of integrating advanced modules like RepVGG, BRA, and C2f_VSS into the architecture to address specific challenges in ECG image analysis. The proposed Mamba-RAYOLO can provide a robust framework for future research and applications in the field of medical diagnostics, offering the potential for more accurate and efficient analysis of medical imaging data.

## Figures and Tables

**Table 1:** Performance comparison of YOLOv7, YOLOv9, and the proposed Mamba-RAYOLO.

Method	Class	Parameters	GFLOPs	Precision	Recall	mAP@0.5	mAP@0.5:0.95
YOLOv7	all			0.507	0.812	0.577	0.562
	ECG HB			0.669	1	0.963	0.936
	History-MI	37,212,738	105.2	0.215	1	0.308	0.295
	MI-ECG			0.602	0.25	0.337	0.326
	Normal-ECG			0.541	1	0.701	0.69
YOLOv9	all			0.909	0.901	0.93	0.889
	ECG HB			0.913	0.95	0.989	0.925
	ECG HBMI-ECG	51,006,520	238.9	0.891	0.86	0.995	0.869
	Normal-ECG			1	0.881	0.745	0.834
				0.832	0.912	0.99	0.929
our method	all			0.947	0.939	0.964	0.959
	ECG HB			0.92	0.87	0.975	0.956
	ECG HBMI	56,496,568	337.7	0.908	0.889	0.89	0.89
	ECG			1	0.998	0.995	0.995
	Normal-ECG			0.961	1	0.995	0.995

**Table 2:** Ablation study of each method in the proposed Mamba-RAYOLO. w/o stands for without.

Module	Parameters	GFLOPs	Precision	Recall
w/o RepVGG	53118008	323.0	0.59	0.61
w/o BRA	51024056	240.0	0.87	0.83
w/o C2f_VSS	53135544	324.0	0.675	0.72

## Data Availability

Data are contained within the article.
